# Reducing Cognitive Effort in Scoring Negotiation Space Using the Fuzzy Clustering Model

**DOI:** 10.3390/e23060752

**Published:** 2021-06-15

**Authors:** Marzena Filipowicz-Chomko, Rafał Mierzwiak, Marcin Nowak, Ewa Roszkowska, Tomasz Wachowicz

**Affiliations:** 1 Faculty of Computer Science, Bialystok University of Technology, Ul. Wiejska 45A, 15-351 Białystok, Poland; m.filipowicz@pb.edu.pl; 2 Faculty of Engineering Management, Poznan University of Technology, Ul. Rychlewskiego 2, 60-965 Poznan, Poland; marcin.nowak@put.poznan.pl; 3 Faculty of Economic and Finance, University of Białystok, Ul. Warszawska 63, 15-062 Bialystok, Poland; e.roszkowska@uwb.edu.pl; 4 Department of Operations Research, University of Economics in Katowice, Ul. 1 Maja 50, 40-287 Katowice, Poland; tomasz.wachowicz@uekat.pl

**Keywords:** decision making, negotiation support, preferences, negotiators’ cognitive profiles, efficient contracts

## Abstract

Negotiation scoring systems are fundamental tools used in negotiation support to facilitate parties searching for negotiation agreement and analyzing its efficiency and fairness. Such a scoring system is obtained in prenegotiation by implementing selected multiple criteria decision-aiding methods to elicit the negotiator’s preferences precisely and ensure that the support is reliable. However, the methods classically used in the preference elicitation require much cognitive effort from the negotiators, and hence, do not prevent them from using heuristics and making simple errors that result in inaccurate scoring systems. This paper aims to develop an alternative tool that allows scoring the negotiation offers by implementing a sorting approach and the reference set of limiting profiles defined individually by the negotiators in the form of complete packages. These limiting profiles are evaluated holistically and verbally by the negotiator. Then the fuzzy decision model is built that uses the notion of increasing the preference granularity by introducing a series of limiting sub-profiles for corresponding sub-categories of offers. This process is performed automatically by the support algorithm and does not require any additional preferential information from the negotiator. A new method of generating reference fuzzy scores to allow a detailed assignment of any negotiation offer from feasible negotiation space to clusters and sub-clusters is proposed. Finally, the efficient frontier and Nash’s fair division are used to identify the recommended packages for negotiation in the bargaining phase. This new approach allows negotiators to obtain economically efficient, fair, balanced, and reciprocated agreements while minimizing information needs and effort.

## 1. Introduction

Negotiation is a complex process in which two or more parties with mixed interests resolve their common decision-making problem [1]. It involves an iterative exchange of the offers and messages between the parties until a satisfying agreement is reached. The subsequent offers submitted to the negotiation table usually follow a predefined negotiation strategy developed in prenegotiation to assure the parties’ goals and aspirations [2,3]. The theory of negotiation defines a list of fundamental conditions for the multilateral problems to be successfully negotiated, one of which is the acceptance of the compromise and intermediate solutions (concessions requitement) [1,4]. Therefore, in the prenegotiations, the parties should be able to jointly define their negotiation problem and formalize the preferences so that the offers to come in bargaining phase could be evaluated, the concessions measured, and their reciprocity confirmed, which could lead them to the identification of commonly accepted satisfying and fair agreement.

For this reason, the theory of negotiation analysis offers a series of support protocols, algorithms and methods that facilitate the negotiators in their prenegotiation activities [5]. The result of their use is a negotiation template (negotiation problem definition) and the scoring system (a system of quantitative scores of the elements of negotiation template). As most negotiations involve many issues, the classic methods from multiple criteria decision aiding (MCDA) are used to design and evaluate such a template [6]. However, real-life negotiations are usually complex and ill-structured processes, in which the information available to negotiators as well as their preferences are incomplete or imprecisely defined (e.g., high price, price of about 90$, low quality, and short delivery time). Moreover, both parties have limited information about the preferences of each other. Therefore, some notions and concepts that accept imprecision and ambiguity in defining the templates and scoring systems were proposed to be incorporated in prenegotiation to build the new support models that use the elements of fuzzy sets theory [7,8]. Unfortunately, the recent experiments on the prenegotiation support efficiency still report on the problems with an adequate template definition and scoring systems design, which is very often linked to the negotiators’ limited cognitive capabilities, insufficient numerical intelligence, or information processing styles that are biased and prone to use heuristics instead of rational reasoning, and their negative impact on negotiation progress and results [9,10,11]. This shows a need for designing new, cognitively easier, and more accessible approaches to support negotiators in preference elicitation and evaluation (individual and mutual) of the negotiation space.

This paper aims to present a novel framework for preference declarations, scoring negotiation offers, and identifying efficient solutions based on a fuzzy clustering model. First, the general method for representing the preferences in negotiations by triangular fuzzy numbers is proposed. Next, the model of preferences is built that derives from the notion of fuzzy clustering. It assumes that, instead of a tiresome evaluation of all elements of the negotiation template, negotiators declare their preferences holistically in terms of complete packages that can be considered as limiting profiles of predefined categories. For such a definition of the preferences, the scoring rules are proposed to cluster any feasible negotiation offer. Some mechanisms for increasing the scoring granularity are also suggested, which reduce the number of offers considered indifferent but simultaneously resign from defuzzification that would question the use of the fuzzy approach at the initial preference elicitation stages. Finally, the methods for searching the Pareto optimal and fair solution in bilateral negotiation are described for our fuzzy clustering model. The usefulness of the proposed approach is shown using a typical example of supply negotiations.

By proposing our framework, we contribute to the theory of negotiation analysis with a novel approach that can be used to organize the prenegotiation preparation activities and the process of suggesting efficient and fair solutions to the negotiating parties. It: (1) accepts uncertainty in the evaluation of negotiation offers that are considered as the limiting profiles in the preference model; (2) reduces the cognitive demand imposed on the parties, not requiring a precise evaluation of all atomic elements of the negotiation template, but operating with a holistic approach and comparisons of complete packages; (3) allows to increase the scoring precision without additional interaction with the negotiator; (4) implements the mechanisms for recommending efficient and fair solutions that may ease the problem of searching of a mutually acceptable agreement.

The paper consists of seven sections. The second section presents an overview of the literature on the fundamental issues related to the decision support in negotiations, i.e., the definition of the negotiation template, its evaluation, determination of the scoring systems, and their use to support the parties in finding the satisfying agreements. Section 3 provides a literature review regarding the approaches used to support negotiators in their prenegotiation tasks related to designing and scoring the negotiation template. It also provides a rationale to design new mechanisms for negotiation support to reduce some limitations of those existing ones. In Section 4, we define some notions and suggest some solutions required to implement the sorting approach to defining and scoring the negotiation template, while in Section 5, the entire framework is introduced. In Section 6, an example of typical supply negotiation is introduced to show how our framework can be used to support the parties in preference elicitation and search of feasible agreements. A sensitivity analysis is also provided to show how the discriminatory power of the scoring system may change while increasing its granularity by changing the number of automatically defined limiting sub-profiles. In the last section, a summary is provided, and conclusions are drawn as well as the future work is outlined.

## 2. Decision Support in Negotiation—Selected Facts 

### 2.1. Negotiation Template, Negotiation Space, and Scoring Systems

Negotiation analysis aims at providing the negotiators with tools that facilitate the entire negotiation process and support them in decision making as well as achieving a mutually satisfying agreement (see [5,12]). The underlying component of such support is the negotiation offer scoring system, which is a numerical representation of the negotiators’ preferences over the elements of the negotiation template. The latter is a detailed description of the negotiation issues and options, i.e., the issues’ feasible resolution levels. Therefore, in the prenegotiation phase, the negotiators are encouraged to work both individually and jointly on developing an accurate structure of their negotiation problem and specify the quantitative systems of their goals, aspirations, and priorities [2,13]. The negotiation template can be defined as the following n + 1 tuple:

(1) $$T=\left\{G,{\left\{O_{i}\right\}}_{i=1}^{n}\right\},$$

where: $G={\left\{g_{i}\right\}}_{i=1}^{n}$ is a set of n negotiation issues, $O_{i}$ is a set of option for *i*th issue, and $\left|O_{i}\right|=l_{i}$ number of options predefined for issue $g_{i}$.

Such a definition of the negotiation template assumes the discrete representation of the negotiation problem. This is, however, a technical simplification only, which does not collide with the continuous nature of some issues (such as price or time of delivery, for instance). In the case of continuous issues or issues with numerous feasible resolution levels, sets $O_{i}$ are assumed to contain only some representatives of all options, namely the salient options [14]. Technically, salient options are such options for which significant changes in the shapes of marginal value functions can be observed, i.e., for which one cannot assume that the priorities may be estimated indirectly based on the evaluation of other neighboring options. 

The aforementioned definition of template T allows formalizing the feasible negotiation space easily. More precisely, the set of feasible negotiation offers (complete packages) may be defined as the Cartesian product of options from $\left\{O_{i}\right\}$, one for each issue i, i.e.,

(2) $$\mathbb{P}=O_{1}\times \ldots \times O_{n}∋P_{k}=\left(p_{k,1},\ldots ,p_{k,n}\right),$$

where: $p_{k,i}\in O_{i}$ denotes an option of issue i in kth package, and $k=1,\ldots ,\;\left|\mathbb{P}\right|,\;\left|\mathbb{P}\right|=l_{1}\cdot l_{2}\cdots l_{n}$.

With the definition of the negotiation space, a problem of supporting negotiators in finding the agreement is technically reduced to the problem of searching space ℙ using the search criteria that address the negotiators’ preferences. Therefore, preferential information should be adequately elicited from the parties during prenegotiation and combined into formal scoring systems that could be used to form such search criteria. Such preferential information is formalized using quantitative ratings that describe the importance of negotiation issues $g_{i}$ and priorities for all feasible resolution levels within each set $O_{i}$. This way, a negotiation offer scoring system is built, which is represented by the following m + 1 tuple

(3) $$S=\left\{W,{\left\{S_{i}\right\}}_{i=1}^{n}\right\},$$

where: $W={\left\{w_{i}\right\}}_{i=1}^{n}$ is a set of weights of negotiation issues, $S_{i}$ is a set of options’ scores (ratings) for *i*th issue, and $\left|S_{i}\right|=l_{i}$.

The entire negotiation space ℙ can be evaluated in view of each negotiator’s scoring system S providing them with information of the relative value of each offer. Hence, the global scoring formula F that allows scoring any package $P_{k}=\left(p_{k,1},\ldots ,p_{k,n}\right)$ from ℙ may be determined using the scoring system in the following way

(4) $$F\left(P_{k}\right)=\sum_{i=1}^{n}w_{i}s_{k,i},$$

where: $s_{k,i}\in S_{i}$ is score rating for option $p_{k,i}^{\;}\in O_{i}$ (k=1,…, |ℙ|; i=1,…,n).

### 2.2. Mutual Evaluation of the Negotiation Space

The negotiators may use the individual scoring system to analyze the profitability of concessions, track the negotiation progress by analyzing the concession paths, visualize the negotiators’ moves on the negotiation history graphs, etc. (see [14,15,16]). However, from the viewpoint of comprehensive negotiation support, the scoring systems of all the parties are recommended to be used jointly by the external helpers to provide the negotiators with suggestions regarding fair, balanced, and reciprocated agreements.

**Figure 1:**
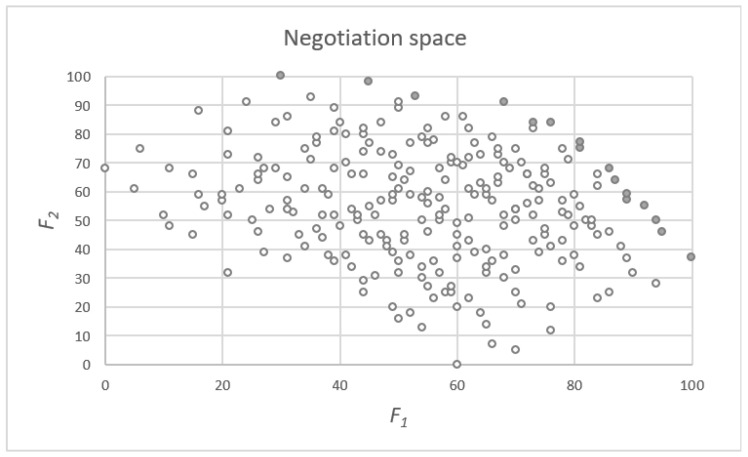
Representation of the negotiation space in the two-dimensional scoring space of both negotiators.

In this paper, we consider bilateral negotiation only; therefore, the negotiation space ℙ can be analyzed in two-dimensional space using scoring systems $S_{1}$ and $S_{2}$ of both parties. In this space, each package is represented by a pair of scores determined according to scoring functions $F_{1}$ and $F_{2}$ (see Figure 1).

In the search for reasonable compromise recommendations, the entire space is limited to the efficient frontier only, i.e., to the subset of Pareto-efficient packages (represented by shaded dots in Figure 1). When the issues are numerical, or the mixes of qualitative options can be logically interpreted, the extreme efficient frontier may be built. Extreme efficiency assumes that any offer may be created as a linear interpolation between any two efficient packages (its rating is assumed to be determined accordingly, as a mix of scores of these two efficient packages).

Having the negotiation space limited to efficient or extreme efficient packages, the third party can easily support the negotiators in identifying a mutually satisfying agreement. If the parties achieved the agreement themselves, but it occurred inefficient, the improvement may be suggested by identifying the nearest packages from the (extreme) efficient frontier to be subject of renegotiation. If the parties are unable to find the compromise themselves, some notion of fair solutions may be applied to find one for them, such as classic Nash fair bargaining solution or other similar (see [17,18,19]). 

## 3. Methods for Scoring the Negotiation Template—Literature Review

### 3.1. Classic Multiple Criteria Decision Aiding Approaches

Since the scoring systems are used to support negotiator individually and jointly (as described in Section 2.2), it is crucial to ensure that they adequately represent the negotiators’ preferences. Therefore, many prenegotiation protocols have been suggested to facilitate the parties’ preference elicitation and template evaluation, mainly implementing the methods from Multiple Criteria Decision Aiding (MCDA) theory. MCDA offers various approaches that can be useful in building a negotiation offer scoring system, as it basically focuses on some similar and more general problems of sorting, rank order, or choosing problematics given multiple evaluation criteria. The majority of MCDA techniques are focused on determining the marginal value functions that describe the negotiators’ within-issue preferences (see, e.g., [20,21,22,23]), assuming the latter are additive and issues are preferentially independent. The preference elicitation may be performed using the original template T or the negotiation space ℙ, which requires the implementation of methods that use the direct aggregation of scores assigned directly or the disaggregation of preferences declared holistically for complete packages.

The SMARTS (Simple Multi-Attribute Ranking Technique) method [24] represents the former ones. It is most frequently used in negotiation analysis [5,25] and requires from the users a direct declaration of crisp ratings and their assignment to the issues and options. It also offers some additional organizational support to declare the issue weights correctly by using the swing weights. SMARTS is also frequently implemented into the prenegotiation protocols of the negotiation support systems (see Inspire [14], ACNF [26], or eNego [15]). The direct rating approach seems quite straightforward; however, it implicitly assumes that parties can declare their preferences quantitatively and precisely without any problems or disturbances. This, in fact, assumes they have prior skills in decision making or went through the training regarding the use of a particular MCDA technique and the meaning and interpretation of the scores obtained. Therefore, some alternative approaches are also implemented that operate with linguistic or quantitative evaluations. The AHP (Analytic Hierarchy Process) method [27] is suggested to help negotiators in bilateral negotiation or consensual group decision problems. It has been applied, for instance, in the Web-HIPRE system [28] to organize the policy for lake regulation or to support bi-negotiation while supplier selection [29]. The TOPSIS (Technique for Order Preference by Similarity to an Ideal Solution) technique was also used to ease the negotiators in defining their preferences [30,31]. The key advantage of TOPSIS-based approaches is that it significantly reduces the workload during preference elicitation as they may automatically evaluate options by implementing some predefined notions of distances. Such an approach was successfully implemented, for instance, in the TOBANS system [32]. However, as it mechanically determines some ratings instead of eliciting them from the negotiators, the scoring systems may not describe the preferences of the negotiators precisely enough. Additionally, some recent experimental studies confirm that determining the scoring systems in negotiation using the approaches that operate with the disaggregated template may involve many problems related to the cognitive capabilities of the negotiators. For example, the use of heuristics or self-serving biases that may heavily impact the quality of the preference elicitation process and lead to the estimation of an imprecise value function was observed [10].

Therefore, alternative approaches are designed to reduce the cognitive burden. They implement holistic judgments that use the principle of preference aggregation–disaggregation and require negotiators to declare their preferences over some subset of complete packages only (e.g., in the form of rank orders or ratings) [33]. This information is disaggregated into the atomic pieces, and hence scores $s_{ij}$ and weights $w_{i}$ can be derived. Examples of such approaches are the UTA (UTilités Additives) method or conjoint analysis [34,35]. The former is considered to be easier and more natural in use for Decision-Makers (DMs) than classic approaches that use the direct rating mechanism or conjoint approach, which requires a precise declaration of rating scores [36,37]. Hence, the UTA-based approach was proposed to support decision analysis in prenegotiation early in the MEDIATOR system and recently in eNego [15,21]. Similar holistic solutions were also proposed that use some hybridized approaches to enhance the scoring system accuracy or reduce the cognitive requirements. An example of such a solution may be MARS (Measuring Attractiveness near Reference Situations) [38], which hybridizes ZAPROS and MACBETH methods, as well as SIPRES, which combines the key elements of the revised Simos’ procedure and the ZAPROS [39].

A few problems with implementing holistic approaches in prenegotiation may occur. First, taking into account the imprecise nature of holistic declarations, it may be unjustified to derive the crisp ratings for options and issues out of them, especially when one realizes that they may depend on some technical parameters of linear programming models required to decompose the preferential information. Second, such a holistic approach requires a predefined subset of alternatives (packages) to be evaluated by the negotiator, and inventing such a subset may be a cognitively demanding task itself. Some experimental studies show that leaving this issue to an inexperienced user may result in quite poor results [40]. This is because the set should be ample enough, and the offers it consists of should be simultaneously easy to rank and diversified. The latter requires them to consist of mixes of options that show how various trade-offs impact the final ranking of offers, which makes them inherently difficult to compare and rank. 

### 3.2. Fuzzy Approaches to Negotiation Support

All the classic approaches described in Section 3.1 assume that the negotiators are to some extent always able to univocally declare their preferences, i.e., distinguish among the evaluated issues and options and set up at least the partial ranking of them. Even if they do not define their preferences quantitatively themselves, the support mechanisms implemented in prenegotiation protocols can transform their qualitative evaluation in the system of crisp cardinal ratings. This assumes, however, that no imprecision occurs in issues and options evaluation while building the scoring systems, nor the negotiators may be unsure about the differences in preferences among the elements of the negotiation template. This is a strong assumption, especially when we consider that many negotiations are representative, i.e., the negotiators are the agents representing their principals, and their understanding of the latter’s preferences may not be ultra-precise and should include uncertainty.

In real life, negotiation is usually a complex and ill-structured problem, e.g., the information available to negotiators and their preferences are incomplete or imprecisely defined (e.g., high price, price of about 90$, low quality, and short delivery time). Moreover, both parties have limited information about the preferences of each other. 

For modelling uncertainly and impression in decision-making problems, several theories offered useful tools, including fuzzy sets [41,42], interval-valued fuzzy sets [43], type 2 fuzzy sets [44,45], interval-valued fuzzy sets [46,47], Dempster–Shafer theory of evidence [48], evidential reasoning [49] rule-base evidential reasoning framework [50], rough sets theory [51], hesitant fuzzy sets theory [52], and soft set theory [53,54]. 

A variety of fuzzy support negotiation models have been proposed in the literature. The fuzzy set concept in determining the negotiators’ preferences was applied in a multi-attribute utility model in a bilateral Negotiation Support System (NSS) by Bui and Sivasankaran [46]. Matos and Sierra [55] analyzed building offers and counteroffers using case-based and fuzzy logic-based strategies. In the case of the fuzzy approach, a set of fuzzy rules has been built to determine the values of the parameters of the negotiation model. Kowalczyk and Bui [56] proposed the Fuzzy e-Negotiation Agents (FeNAs) model for autonomous multi-issue negotiation in e-commerce. In this model, limited common knowledge, imprecise information, and preferences are represented by fuzzy and linguistic offers that describe the level of satisfaction of an agent with potential solutions.

In another paper, Kim [57] described a Fuzzy and AHP (Analytic Hierarchy Process)-based Negotiation Support Mechanism (FAHP-NEGO) to support the negotiation in Electronic Commerce (EC). The fuzzy membership function is used for representing the buyer and seller cognition for issues such as quantity, price, quality, and delivery, while the AHP procedure measures preferences and satisfaction from the offer and the counteroffer. Lai and Lin [58] proposed a framework for modeling multi-issue multilateral agent negotiation in e-business using fuzzy constraints. Raeesy et al. [59] presented a fuzzy-based model for negotiation with qualitative values. In the double protocol, both fuzzy and crisp values can be proposed to the opponent. They concluded that “exchanging fuzzy values as offers leads to a more flexible negotiation”. Zuo and Sun [60] presented a model to support bilateral agent negotiation using fuzzy logic. Roszkowska and Burns [61] proposed the conceptualization of fuzzy bargaining games in the context of conditions of agreement, satisfaction, and equilibrium. Tsai and Chou [62] proposed a fuzzy multi-attribute matching mechanism for sealed-bid and single round auctions and a Fuzzy Negotiation System (FNS) to evaluate this mechanism. The matching system is constructed using triangular fuzzy numbers. In the paper, Zandi and Tavana [63] presented a fuzzy e-negotiation support system based on cooperative multi-criteria game theory. The model joins three elements: a fuzzy cooperative multi-criteria game theory, Internet technologies, and an e-negotiation support system for new product development. The fuzzy sets are used to deal with imprecise and incomplete information in the negotiation process. Yang and Luo [64] proposed a multi-demand negotiation model with fuzzy concession strategies. A multi-demand negotiation model implemented fuzzy rules obtained by psychological experiments were described by [65]. In two papers [7,66], models predictive control based on fuzzy negotiation were proposed. 

Roszkowska and Wachowicz [67] proposed the fuzzy multi-criteria analysis for negotiation support. The membership function is used to express the negotiator’s imprecise preferences, where options are poorly defined and cannot be described with conventional quantitative terms. Fu et al. [68] proposed a Fuzzy System (FS) approach to provide a negotiation price boundary by learning from available historical data. 

As defining preferences over the template or negotiation space elements may be cognitively demanding [69], the linguistic approach is also implemented in various MCDA algorithms and often combined with the fuzzy approach to support the negotiators [70,71]. To deal with the imprecise and vague judgment of negotiators, the fuzzy versions of TOPSIS [8,70,71] or SAW (Simple Additive Weighing) [70,71] algorithms were proposed. The options of negotiation issues are described by the fuzzy or ordered fuzzy numbers, mainly triangular or trapezoidal.

In this paper a novel framework of scoring negotiation offers based on a fuzzy clustering model, were the negotiation option as well as negotiation packages are represented by triangular fuzzy numbers is proposed. 

## 4. Using Sorting Approach and Limiting Profiles to Evaluate the Negotiation Space

Although the negotiation analysis currently offers a variety of decision support approaches to facilitate the prenegotiation preparation and evaluation of the negotiation template, they still reveal some shortages. Some of them enforce the negotiators to operate with precise declarations of the strength of preferences for the disaggregated templates, which is time-consuming and unnatural to many negotiators, as they are forced to evaluate the options regardless of the potential offers they constitute. Some others allow for holistic declarations but require series of countless and tiresome comparisons of ample reference sets to define the preferences at acceptably accurate level. Alternatively, they allow using imprecise preference declarations employing linguistic scales and accompanied grey or fuzzy numbers, but operate with the numerical equivalents a priori defined for these scale without any reflection on how the negotiators may perceive these scales and how they interpret the verbal etiquettes from these scales in terms of quantitative relationships. Additionally, when the imprecision of evaluation is allowed, and fuzzy numbers are used to describe the preferences, they are eventually very often defuzzified to allow for comparisons between the offers and provide the negotiators with a univocal recommendation regarding the offer quality and the scale of concessions it requires. Therefore in this paper, we propose an alternative approach that, on the one hand, would allow negotiators to imprecisely and holistically declare their preferences over the subset of feasible negotiation space, but simultaneously will allow expanding this preference information over the entire negotiation space and use these imprecisely preferences to measure the differences between the quality of the offer and suggest the potential agreement’s improvements or fair compromises without any unjustified aggregations to their scalar and crisp form. The underlying element of this approach is a change in the perception of the problem under considerations, i.e., it replaces the problem of ranking the alternatives with the problem of their sorting [72]. 

In this approach, it is assumed that instead of determining the ratings of alternatives, they need to be assigned to the predefined categories of quality. Hence, instead of defining the set of reference alternatives out of the ranking of which the value functions are determined, it is enough to define the examples of offers that will be considered as the limiting profiles of the predefined categories. Such an approach seems quite universal, as it may be applied to basically any core MCDA technique used later to assign the offers into the classes (see [73,74,75]). More importantly, such examples of limiting profiles for subsequent categories are easier to define than reference alternatives in ranking the problem, as they usually are represented by a series of Pareto-dominating alternatives (e.g., the limiting profile of the best and good categories consists of options better than the limiting profile for categories good and average, for every single issue). Such an approach has already been suggested for classifying negotiation offers using the outranking relations and ELECTRE TRI method [76,77]. 

Despite the fact that the sorting-based approach seems promising in prenegotiation offer evaluation (as potentially reducing the cognitive demand from the negotiators), some issues should be addressed and solved before applying it to the process of scoring the negotiation template. Two issues are the most important. First, how to increase the granularity of the evaluation of the offers, here limited to some (at most a few only) ordered categories that would allow differentiating among all (or at least the majority of) the offers within ℙ. Below, we propose an original solution to this issue.

**Figure 2:**
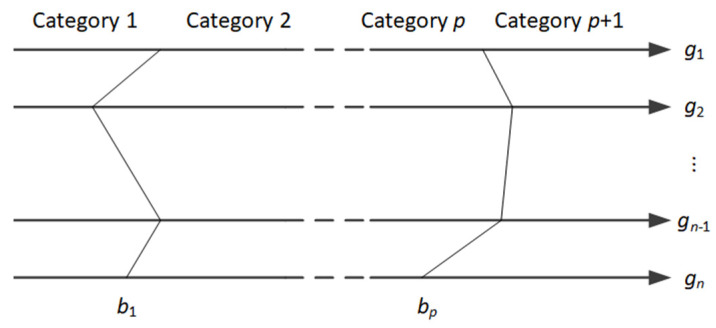
Categories and profiles in negotiation space T.

Let us assume that for negotiation space ℙ, the negotiator defines the set of limiting profiles $B={\left\{B_{h}\right\}}_{h=1}^{p}$ ($B_{h}=\left(b_{h,1},\ldots ,b_{h,n}\right)$) defined in the form of complete packages built out of the options defined in T. These profiles allow defining the p+1 categories of the negotiation offer in a way that the profile $B_{h}$ is considered to be the upper limit of the category $C_{h}$ and the lower limit of the category $C_{h+1}$. We assume that the categories are ordered, i.e., the higher the category index, the more preferred offers it contains. We may further assume, without loss of generality, that the negotiator’s preferences increase with an increase of the value of each issue G (see Figure 2).

Using the aforementioned definition of categories and profiles, the classification rule may be defined, which will allow to assign any offer $P_{k}$ from ℙ to one of the predefined categories $C_{h}$:

(5) $$C\left(P_{k}\right)=\{\begin{array}{ll} C_{1} & \mathrm{if}\;P_{k}≺B_{1}\\ C_{h} & \mathrm{if}\;P_{k}≽B_{h-1}\wedge P_{k}≺B_{h}\\ C_{p+1} & \mathrm{if}\;P_{k}≽B_{p} \end{array}$$

where: ≻(≽) is a binary relation allowing to consider if one offer is better (better or equal) to another according to the negotiator’s preference system. The philosophy that stands behind the process of confirming such a relation for any two alternatives depends on the MCDA technique applied.

However, to be able to provide the negotiators with mutual support as described in Section 2.2, the scores should be assigned to each offer in ℙ. In this approach, we assume that a score each offer $P_{k}$ receives is equivalent to the rank of the category it was assigned to according to the rule (5):

(6) $$F\left(P_{k}\right)=\left\{h:C\left(P_{k}\right)=C_{h}\right\}.$$

Hence, no scoring system as defined by S will be determined, yet the scoring function (6) is formulated as the equivalent of Formula (3).

**Figure 3:**
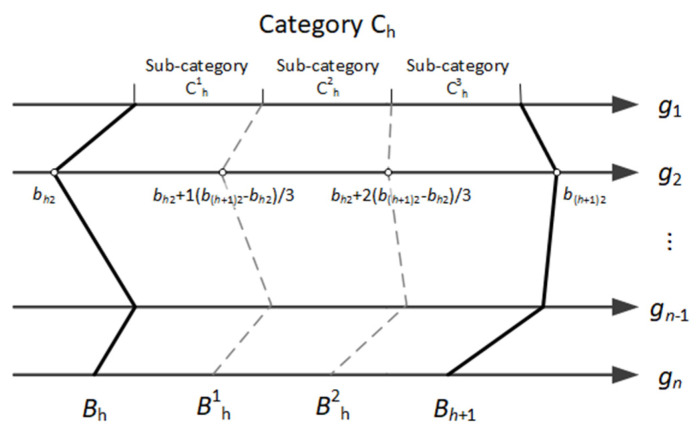
Sub-categories and their limiting profiles defined additionally in negotiation space T.

Naturally, the small number of categories results in the narrow evaluation scale and may make many offers to be indistinguishable in the sense of preferences. We may, however, increase the evaluation scale in our approach by implementing the same notion of interpolation that is classically used to determine the scores for options other than salient in traditionally defined template T. If a larger evaluation scale is needed, we propose to divide each category $C_{h}$ it into $n_{h}$ sub-categories of equal dimensions. Each sub-category is defined by new profiles that are equidistant from each other and lay between the lower and upper limiting profiles of this category $C_{h}$. They can be determined automatically by linear interpolation between the issues’ values of neighboring profiles $B_{h}$ and $B_{h+1}$ that define this category. The upper-limit profile of each sub-category s ($s=1,\ldots ,n_{h}$) can be determined as $B_{h}^{s}=(b_{h,1}^{s},\ldots ,\;b_{h,n}^{s})$, where:

(7) $$b_{h,i}^{s}=b_{h,i}+s\frac{b_{\left(h+1\right),i}-b_{h,i}}{n_{h}}$$

for i=1,…,n. The upper limit profile for the sub-category $n_{h}$ is $B_{h}$. The visualization of this approach is shown in Figure 3.

The only remaining issue that should be considered now is how the preferences of the negotiators should be captured to allow the classification rule (5) to assign each offer into a corresponding category and sub-category. In the following subsection, the issue of implementing the linguistic scales to define the categories decoding them into fuzzy numbers describing the performance of each limiting profile within each issue is introduced.

## 5. Procedure of Scoring Negotiation Space Using the Fuzzy Clustering Model

### 5.1. Fuzzy Numbers in Scoring the Limiting Profiles 

The preliminary definitions of fuzzy sets, fuzzy numbers, fuzzy operations and fuzzy preferences are presented below.

*Fuzzy number.* A fuzzy number is defined as a fuzzy subset of the universe of discourse ℜ that is both convex and normal. The most commonly used form of fuzzy numbers is Triangular Fuzzy Numbers (TFNs).

*Triangular fuzzy number.* Triangular fuzzy number $\tilde{A}$ is defined on ℜ as a fuzzy subset with the membership function $\mu_{\tilde{A}}\left(x\right)$:

(8) $$\mu_{\tilde{A}}\left(x\right)=\{\begin{array}{cc} 0 & \mathrm{for}\;x<a_{l}\\ \frac{x-a_{l}}{a_{m}-a_{l}} & \;\;\;\;\;\mathrm{for}\;a_{l}\leq x\leq \;a_{m}\\ \frac{x-a_{u}}{a_{m}-a_{u}} & \;\;\;\;\;\mathrm{for}\;a_{m}<x\leq \;a_{u}\\ 0 & \mathrm{for}\;x>a_{u} \end{array},$$

Then, TFN $\tilde{A}$ can be represented by $\left(a_{l},a_{m},a_{r}\right)$, where: $a_{l}$ is the left threshold value, $a_{m}$—the midpoint, and $a_{r}$—the right threshold value.

*Operations on TFNs.* For any given two TFNs $\tilde{A}=\left(a_{l},a_{m},a_{r}\right)$ and $\tilde{B}=\left(b_{l},b_{m},b_{r}\right)$ and a positive real number r, the main operations of fuzzy numbers $\tilde{A}$ and $\tilde{B}$ can be expressed as follows:

(9) $$\mathrm{addition}:\;\tilde{A}⊕\tilde{B}=\left(a_{l}+b_{l},a_{m}+b_{m},a_{r}+b_{r}\right),$$

(10) $$\mathrm{multiplication}\;\mathrm{by}\;a\;\mathrm{scalar}:\;\tilde{A}⊗r=\left(a_{l}r,a_{m}r,a_{u}r\right),$$

(11) $$\mathrm{multiplication}:\;\tilde{A}⊗\tilde{B}≅\left(a_{l}b_{l},a_{m}b_{m},a_{r}b_{r}\right).$$

*The fuzzy weights and normalization formula*. We adopt the notion of fuzzy weights proposed by Wang and Elhag [78]. Let $G={\left\{g_{i}\right\}}_{i=1}^{n}$ be the set of criteria. Set $W=\left\{\tilde{w_{1}},\ldots ,\tilde{\;w_{n}}\right\}$ constitutes the set of fuzzy criteria weights, where: $\tilde{w_{i}}=\left(w_{i,l},w_{i,m},w_{i,u}\right)\;(i=1,\;2,\ldots ,n$) expresses the importance degrees of $g_{i}$, if all $\tilde{w_{i}}$ are positive fuzzy numbers and

(12) $$\sum_{j=1}^{n}w_{j,m}=1,$$

(13) $$w_{i,l}+\sum_{j=1,i\neq j}^{n}w_{j,u}\geq 1,$$

(14) $$w_{i,u}+\sum_{j=1,i\neq j}^{n}w_{j,l}\leq 1,$$

for every i=1, 2,…,n.

Let us assume that the decision maker (DM) assigns to each criterion $G={\left\{g_{i}\right\}}_{i=1}^{n}$ individual positive fuzzy weights ${\tilde{r}}_{i}=\left(r_{i,l},r_{i,m},r_{i,u}\right)$ representing the importance of the criteria (i=1, 2,…,n). To normalize the positive fuzzy numbers ${\tilde{r}}_{i}=\left(r_{i,l},r_{i,m},r_{i,u}\right)$, where: $r_{i,l}>0,$ we apply the following formula [78]:

(15) $$\tilde{w_{i}}=\left(\frac{r_{i,l}}{\;r_{i,l}+\sum_{i=1,j\neq k}^{n}r_{j,u}},\;\frac{r_{k,m}}{\;\sum_{j=1}^{n}r_{j,m}},\;\frac{r_{k,u}}{\;r_{k,u}+\sum_{j=1,j\neq k}^{n}r_{j,l}}\right)\;(i=1,\;2,\ldots ,n).$$

*Fuzzy approximation procedure.* Let $\tilde{x}=\left(x_{l},\;x_{m},\;x_{r}\right)$,$\;\tilde{y}=\left(y_{l},\;y_{m},\;y_{r}\right)$ be fuzzy representations of negotiation options x,y described by real numbers, respectively. Then:

(a) for x<z<y, we have $\tilde{z}=\left(z_{l},\;z_{m},\;z_{r}\right),$ where:
  (16) $$z_{\alpha }=\left(y_{\alpha }-x_{\alpha }\right)\frac{y-z}{y-x}+x_{\alpha }\;(\alpha =\left\{l,\;m,\;r\right\}),$$
(b) for y<z<x, we have $\tilde{z}=\left(z_{l},\;z_{m},\;z_{r}\right)$, where:
  (17) $$z_{\alpha }=-\left(y_{\alpha }-x_{\alpha }\right)\frac{y-z}{y-x}+y_{\alpha }\;(\alpha =\left\{l,\;m,\;r\right\}).$$

*Comparison and rank ordering fuzzy numbers.* To compare two fuzzy numbers, we used the fuzzy preference relation with the membership function representing the preference degree proposed by Wang [79]. This approach seems more reasonable because defuzzification on ranking fuzzy numbers does not take into account the preference degree between two fuzzy numbers; therefore, some information may be lost.

Let $\tilde{A}=\left(a_{l},b_{m},c_{r}\right)$ and $\tilde{B}=\left(b_{l},b_{m},b_{r}\right)$ be two TFNs. A fuzzy preference relation P is a fuzzy subset of ℜxℜ with membership function $\mu_{P}\left(\tilde{A},\;\tilde{B}\right)$ representing the preference degree of A over B defined by [79] as follows:

(18) $$\mu_{P}\left(\tilde{A},\;\tilde{B}\right)=\frac{1}{2}\left(\frac{\left(a_{l}-b_{r})-2(a_{m}-b_{m})+(a_{r}-b_{l}\right)}{2\|T\|}+1\right),$$

where:

(19) $$\|T\|=\{\begin{array}{c} \frac{\left(\;t_{l}^{+}-t_{r}^{-}\;\right)+2\left(t_{h}^{+}-t_{h}^{-}\right)+\left(t_{r}^{+}-t_{l}^{-}\;\right)}{2}\;\mathrm{if}\;t_{l}^{+}\geq t_{r}^{-}\;\\ \frac{\left(\;t_{l}^{+}-t_{r}^{-}\;\right)+2\left(t_{m}^{+}-t_{m}^{-}\right)+\left(t_{r}^{+}-t_{l}^{-}\;\right)}{2}+2\left(t_{r}^{-}-t_{l}^{+}\right)\;\mathrm{if}\;t_{l}^{+}<t_{r}^{-} \end{array},$$

$t_{l}^{+}=\max\left(a_{l},\;b_{l}\right),$ $t_{m}^{+}=\max\left(a_{m},\;b_{m}\right),$ $t_{r}^{+}=\max\left(a_{r},\;b_{r}\right),$ $t_{l}^{-}=\min\left(a_{l},\;b_{l}\right)$, 

$t_{m}^{-}=\min\left(a_{m},\;b_{m}\right),$ $t_{r}^{-}=\min\left(a_{r},\;b_{r}\right)$.

We say that $\tilde{A}$ is preferred to $\tilde{B}$ if $\mu_{P}\left(\tilde{A},\;\tilde{B}\right)>\frac{1}{2}$. On the other hand, A is equal to B if $\mu_{P}\left(\tilde{A},\tilde{B}\right)=\frac{1}{2}$.

*The notion of fuzzy scoring system represented by TFN.* Let $T=\left\{G,{\left\{O_{i}\right\}}_{i=1}^{n}\right\}$ be a negotiation template (see Formula (1)). Then, the negotiation fuzzy offer scoring system can be represented by the following n + 1 tuple

(20) $$\tilde{S}=\left\{W,{\left\{{\tilde{S}}_{i}\right\}}_{i=1}^{n}\right\},$$

where: W is a fuzzy set of weights of negotiation issues and ${\tilde{S}}_{i}$ is a set of representation options from $S_{i}$ by TFNs.

In this way, the fuzzy representation of package $P_{k}=\left(p_{k,1}^{\;},\ldots ,p_{k,n}\right)$ from ℙ has the form ${\tilde{P}}_{k}=\left({\tilde{p}}_{k,1}^{\;},\ldots ,{\tilde{p}}_{k,n}\right)$, where ${\tilde{p}}_{k,i}^{\;}\in {\tilde{S}}_{i}$ (i=1,…,n) and the final fuzzy score of package $P_{k}$ is calculated as follows:

(21) $$\tilde{F}\;\left(P_{k}\right)=\sum_{i=1}^{n}{\tilde{w}}_{i}⊗{\tilde{p}}_{k,i}^{\;},$$

where: ${\tilde{p}}_{k,i}^{\;}$ is the fuzzy value of the kth package with respect to the ith criterion and ${\tilde{w}}_{i}$ is the weight of the ith criterion. 

### 5.2. Algorithm of Scoring Negotiation Space Using the Fuzzy Clustering Model 

**Figure 4:**
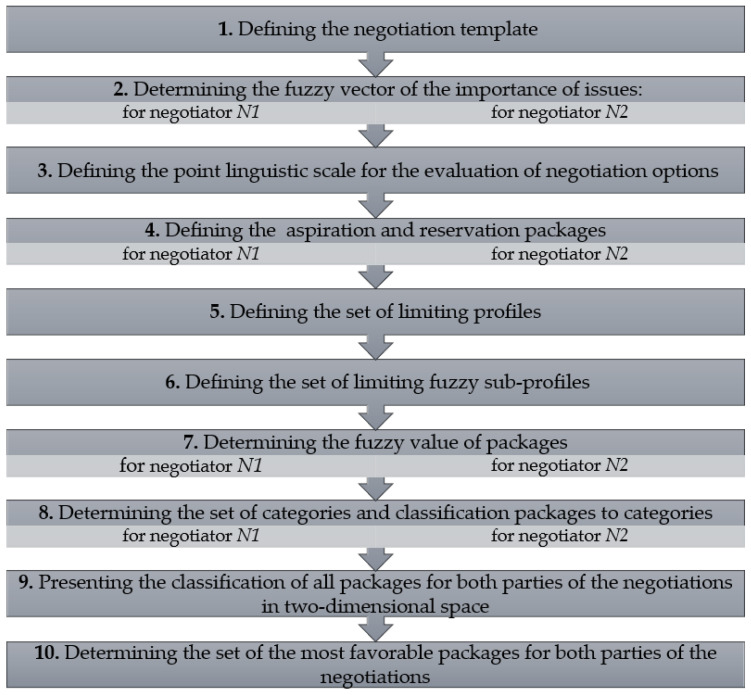
Scheme of the procedure of scoring negotiation space.

The procedure of scoring negotiation space in bilateral negotiation using the fuzzy clustering model is presented in the Figure 4.

The steps of algorithm are as following: 

**Step 1.** Defining the negotiation template, i.e., set of negotiation issues and the negotiation space.

Let us assume that N1 and N2 denote the negotiator one and two, respectively. The negotiation template for both parties has the form $T=\left\{G,{\left\{O_{i}\right\}}_{i=1}^{n}\right\}$ as described in Formula (1).

For each party, we have $G=Be^{Q}\cup Co^{Q}$, where: $Be^{Q}$ is the set of benefit criteria, $Co^{Q}$ is the set of cost criteria for Q, where: Q={N1,N2}. In the case of quantitative issues, we assume that they are monotonic. 

Let $\mathbb{P}=O_{1}\times \ldots \times O_{n}∋P_{k}=\left(p_{k,1}^{\;},\ldots ,p_{k,n}\right)$ as was noted in Formula (2), where: $p_{k,i}\in O_{i}$ denotes an option of issue i in kth package, $k=1,\;\ldots ,\;\left|\mathbb{P}\right|,\;\left|\mathbb{P}\right|=l_{1}\cdot l_{2}\cdots l_{n}$.

**Step 2.** Determining the fuzzy vector of the importance of issues for negotiators N1,N2.

Let $W^{Q}={\left\{{\tilde{w}}_{i}^{Q}\right\}}_{i=1}^{n}$ be the set of fuzzy weights for issues from the set G for Q={N1,N2} (see Formula (15)). 

The weights can be determined using a linguistic evaluation scale. Let *LT* denote the set of linguistic terms, L={1,…,s}—the set of linguistic labels. An example of such linguistic scale for s=9 is presented in Table 1. Note, however, that some other alternative methods may be used here to elicit the issue importance, for instance, when the cognitive limitations of negotiators require deeper or more transparent facilitation of the process of preference impartation. 

**Step 3.** Defining the s—point linguistic scale for the evaluation of negotiation options represented by TFNs (s≥3).

An example of a 7-point linguistic scale is presented in Table 2.

**Step 4.** Defining the aspiration $P_{as}^{Q}$ and reservation package $P_{res}^{Q}$ for negotiators N1,N2.

Let $P_{as}^{Q}=\left(p_{as,1}^{Q},p_{as,2}^{Q},\ldots ,p_{as,n}^{Q}\right)$ represent the aspiration package and $P_{res}^{Q}=\left(p_{res,1}^{Q},p_{res,2}^{Q},\ldots ,p_{res,n}^{Q}\right)$ reservation package for Q={N1,N2}.

Determining the levels of aspiration and reservation is consistent with the assumptions of the negotiation analysis [1,5]. If the quantitative criterion is of the profit type, the aspiration level can be designated as the maximum option value, and the reservation level as the minimum option value of the given criterion. In the case of the quantitative cost type criterion, we proceed the other way round. If the criterion is qualitative, then each option is assigned a linguistic term according to the adopted scale in Step 3. Then, the level of aspiration and reservation is assigned with the highest and the lowest linguistic label, respectively, used to order the options for this criterion.

**Step 5.** Defining the set of limiting profiles. 

The set of limiting profiles is defined in the form of complete packages and their fuzzy representation based on the linguistic scale (chosen in Step 3). The sets of limiting profiles $B^{Q}$ are built by negotiators separately out of the options evaluated according to the subset of linguistic terms (*SLT*) from *LT,* Q={N1,N2}. Moreover, let *SL* be the set of linguistic labels describing linguistic terms from *SLT.* We assumed that the linguistic terms representing reservation and aspiration packages are in *SLT*. 

Let:

(22) $$B^{Q}={\left\{\;B_{z}^{Q}:\;B_{z}^{Q}=\left(b_{z,1}^{Q},\ldots ,b_{z,n}^{Q}\right)\;\mathrm{for}\;z\in SL\right\}}_{\;}^{\;},$$

where: $b_{z,i}^{Q}\in O_{i}$ is an option with the z—linguistic label from SL for ith issue (i=1, 2, …, n), and Q={N1,N2}. 

Let us note that $B_{1}^{Q}=P_{res}^{Q},\;\;B_{s}^{Q}=P_{as}^{Q}$. Moreover, $B_{u}^{Q}$ is less preferred than $B_{v}^{Q}$ ($B_{u}^{Q}≺B_{v}^{Q})$ if u<v, (u,v ∈SL). Now, define the set of fuzzy limiting profiles as follows:

(23) $${\tilde{B}}^{Q}=\left\{{\tilde{B}}_{z}^{Q}:\;{\tilde{B}}_{z}^{Q}=\left({\tilde{b}}_{z,1}^{Q},\ldots ,{\tilde{b}}_{z,n}^{Q}\right)\;\;\mathrm{for}\;z\in SL\right\},$$

where: ${\tilde{B}}_{z}^{Q}$ is the fuzzy profile represented by zth label options and ${\tilde{b}}_{z,i}^{Q}$—fuzzy representation of the zth label options for the ith criterion. 

**Step 6.** Define the set of limiting fuzzy sub-profiles. 

Let h be the number of divisions. The fuzzy sub-profiles ${\tilde{B}}_{z,h}^{Q}$ are determined in the following way:

(24) $${\tilde{B}}_{z,0}^{Q}={\tilde{B}}_{z\;}^{Q}\;\;\mathrm{for}\;z\in SL$$

(25) $${\tilde{B}}_{z,r}^{Q}=\left(\frac{h-r}{h}⊗{\tilde{B}}_{z}^{Q}\right)⊕\left(\frac{r}{h}⊗{\tilde{B}}_{z+1}^{Q}\right)\;\mathrm{for}\;r=1,\ldots ,h-1,z\in SL\backslash \left\{s\right\}.$$

In this way, we have λ=|SL|+(h−1)(|SL|−1) fuzzy sub-profiles. The fuzzy sub-profiles ${\tilde{B}}_{z,0}^{Q}$ are determined under a subjective negotiator evaluation, while ${\tilde{B}}_{z,r}^{Q}$ for r=1,…,h−1 are technically calculated.

**Step 7.** Determining the fuzzy value of packages from the set $\mathbb{P}\backslash B^{Q}\;\left(Q=N1,\;N2\right)$.

Let $P_{k}^{Q}=\left(p_{k,1}^{Q},p_{k,2}^{Q},\ldots ,p_{k,n}^{Q}\right)\in \mathbb{P}\backslash B^{Q}$, for $k=1,\;\ldots ,\;\left|\mathbb{P}\right|,\;\left|\mathbb{P}\right|=l_{1}\cdot l_{2}\cdots l_{n}.$ The fuzzy representation ${\tilde{p}}_{k,i}^{Q}$ of option $p_{k,i}^{Q}$ is determined according to Formula (16) if $b_{z,i}^{Q}<p_{k,i}^{Q}<b_{z+1,i}^{Q}$ and Formula (17) if $\;b_{z+1,i}^{Q}<p_{k,i}^{Q}<b_{z,i}^{Q}$, for some z, z+1∈ SL. Then, the fuzzy score $\tilde{F}(P_{k}^{Q})$ of package $P_{k}^{Q}\in \mathbb{P}\backslash B^{Q}$ is calculated by using Formula (21). 

**Step 8.** Determining the set of categories $C^{Q}$ based on the set of limiting sub-profiles $B^{Q}$ and classifying packages to categories. 

Let $C^{Q}=\left\{C_{z,r}^{Q}:\;z\in SL\backslash \left\{s\right\},\;r=1,\ldots ,\;h-1\;\right\}\cup \left\{C_{z,r}^{Q}:\;z\in SL,\;r=0\;\right\},$ where: $C_{z,r}^{Q}=\{P_{k}^{Q}\in \mathbb{P}:\mu_{P}\left(\tilde{F}(P_{k}^{Q}),\tilde{F}(B_{z,r}^{Q})\right)\geq 0.5\;\wedge \;\mu_{P}\left(\tilde{F}(P_{k}^{Q}),\tilde{F}(B_{z,r+1}^{Q})\right)<0.5\}$.

**Figure 5:**
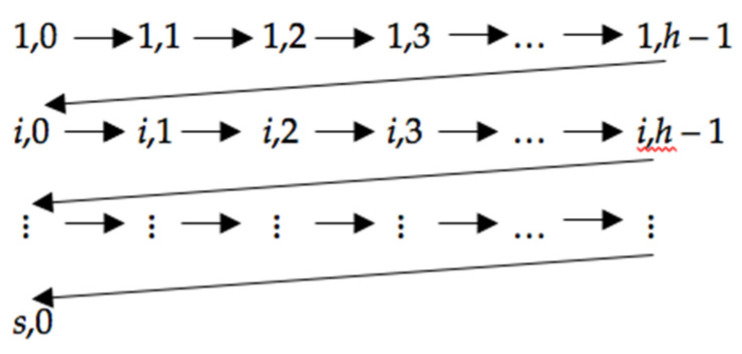
Assigning numbers *z*,*r* consecutive natural numbers. (Note: *i* is the next label after 1 from *SL*).

Moreover, note that the set of pairs z,r is an ordered finite set, so each pair can be assigned a natural number t in the following way (see Figure 5).

Then, $C_{z,r}^{Q}=C_{t}^{Q}$, where: t denotes the number of categories and t=1, 2, …, λ and $P_{k}^{Q}\;\in C_{t}^{Q}$ if and only if $\mu_{P}\left(\tilde{F}(P_{k}^{Q}),\tilde{F}(B_{t}^{Q})\right)\geq 0.5\;\wedge \;\mu_{P}\left(\tilde{F}(P_{k}^{Q}),\tilde{F}(B_{t+1}^{Q})\right)<0.5\}$, $k=1,\;\ldots ,\;\left|\mathbb{P}\right|,\;\left|\mathbb{P}\right|=l_{1}\cdot l_{2}\cdots l_{n}$.

**Step 9**. Presenting the obtained classification of all packages for both parties of the negotiations in two-dimensional space as the points (t(N1), t(N2)), where: t(N1), t(N2) denoted the number of the cluster obtained for the same package in the case of the negotiators N1 and N2.

**Step 10.** Determining the set of the most favorable packages for both parties of the negotiations. 

Packages, which are on the efficient frontier and which are fair, must fulfill the following two conditions:

1. t(N1), t(N2)→max
2. |t(N1)−t(N2)|→min

The most desirable situation is when |t(N1)−t(N2)|=0, therefore, in a case when the given package was assigned to the same category by both negotiation parties.

## 6. Numerical Example

To verify the theoretical approach proposed in Section 5.2, the numerical example, based on the negotiation case found in the eNego system [15], will be presented. In Step 1 of the algorithm the negotiation template is defined. We consider bilateral negotiation. One party is the bicycle producer (*N*1), and the second one is the parts supplier (*N*2). Their aim is to negotiate a new contract for the delivery of rear-wheel gears. Four issues that are taken into account (price ($g_{1}$), delivery time ($g_{2}$), payment ($g_{3}$), and returns conditions ($g_{4}$)) and for each of them, the sets of defined options ($O_{i}$) made the following negotiation template (see Table 3).

Considering all combinations within the issues, we obtained the set of all packages ℙ, where |ℙ|=5580. Examples of packages $P_{k}=\left(p_{k,1}^{\;},p_{k,2}^{\;},p_{k,3},p_{k,4}\right)\in \mathbb{P},$ where k=1, 2, …, 5580) are presented in Table 4.

The levels of realizations of issues $g_{1},\;g_{2},g_{3}$ are described by means of numerical values. For the recipient (*N*1), the issues $g_{1}$,$g_{2}$ are the cost issues, while $g_{3}$ is the profit. From the supplier’s (*N*2) point of view, the criteria $g_{1}$,$\;g_{2}$ are the profit issues, while $g_{3}$ is the cost issue.

For the returns conditions ($g_{4}$), the negotiators provide the linguistic evaluations represented by TFNs according to Table 2. The results are presented in Table 5.

In Step 2, the negotiators *N*1 and *N*2 determine the fuzzy weights of the importance of issues using the 9-point scale linguistic evaluation from Table 1. Table 6 and Table 7 present the fuzzy weights as well as the fuzzy normalized weights for both negotiators using Formula (15). 

In Step 3, the 7-point linguistic scale was chosen (see Table 2) represented by TFNs for evaluation options. 

In Step 4, the negotiators define the aspiration packages as $P_{as}^{N1}=\left(10,\;14,\;60,E\right),\;P_{as}^{N2}=\left(25,\;90,\;1,A\right),$ and the reservation packages as $P_{res}^{N1}$= (25, 90, 1,A ), $P_{res}^{N2}$ = (10, 14, 60, D) for *N*1, *N*2, respectively. The reservation packages are evaluated as very poor, and the aspiration ones as very good. 

Moreover, in Step 5, the negotiators are asked to choose these options for every criterion which they evaluate as poor, fair, medium good, and good. The distinguished set of linguistic terms *SLT* = {very poor, poor, fair, medium good, good, very good} is represented by the set of linguistic labels *SL* = {1, 2, 4, 5, 6, 7}, respectively. Then, we obtained the following packages with the zth linguistic labels (z∈SL) in order from the least preferred to the most preferred for recipient (*N*1): $B_{1}^{N1}=P_{res}^{N1},\;B_{2}^{N1}=\left(22,\;75,\;7,A\right),\;B_{4}^{N1}=\left(21.5,\;45,\;14,D\right),B_{5}^{N1}=\left(20,\;30,\;30,C\right),\;B_{6}^{N1}=\left(18,\;21,\;45,\;E\right),\;B_{7}^{N1}=P_{as}^{N1}$, and for supplier (*N*2): $B_{1}^{N2}=P_{res}^{N2},B_{2}^{N2}=\left(12,\;21,\;45,D\right),\;B_{4}^{N2}=\left(15,\;30,\;30,C\right),B_{5}^{N2}=\left(17.5,\;45,\;14,\;B\right),\;B_{6}^{N2}=\left(20,\;75,\;7,\;A\right),\;B_{7}^{N2}=P_{as}^{N2}.$ These packages defined the sets of limiting profiles $B^{N1}=\left\{B_{z}^{N1}:\;B_{z}^{N1}=\left(b_{z,1}^{N1},b_{z,2}^{N1},b_{z,3}^{N1}b_{z,4}^{N1}\right)\right\}$ and $B^{N2}=\left\{B_{z}^{N2}:B_{z}^{N2}=\left(b_{z,1}^{N2},b_{z,2}^{N2},b_{z,3}^{N2},\;b_{z,4}^{N2}\right)\right\},$ where z∈{1, 2, 4, 5, 6, 7} for two negotiators separately. Every option $b_{z,i}^{Q}$ in the package of limiting profiles has its fuzzy representation: ${\tilde{b}}_{1,i}^{Q}=\left(0,\;0,\;1\right)$, ${\tilde{b}}_{2,i}^{Q}=\left(0,1,\;3\right)$ ${\tilde{b}}_{4,i\;}^{Q}=\left(3,\;5,\;7\right)$, ${\tilde{b}}_{5,i\;}^{Q}=\left(5,\;7,\;9\right)$, ${\tilde{b}}_{6,i\;}^{Q}=\left(7,\;9,\;10\right)$, and ${\tilde{b}}_{7,i\;}^{Q}=\left(9,\;10,\;10\right)$, where: i=1, 2, 3, 4 and Q={N1, N2}.

In Step 6, by using Formula (25) for arbitrarily taken h=5, twenty-six limiting fuzzy sub-profiles (${\tilde{B}}_{z,r}^{Q}\cup {\tilde{B}}_{z}^{Q},$ where z∈{1, 2, 4, 5, 6, 7}, r∈{1, 2, 3, 4}) for each negotiator are determined. The set of all fuzzy sub-profiles for *N*1 is as follows:

{(0.00, 0.00, 1.25), (0.00, 0.20, 1.75), (0.00, 0.40, 2.25), (0.00, 0.60, 2.75), (0.00, 0.80, 3.25), (0.00, 1.00, 3.75), (0.47, 1.80, 4.75), (0.94, 2.60, 5.75), (1.41, 3.48, 6.75), (1.89, 4.20, 7.75), (2.36, 5.00, 8.75), (2.67, 5.40, 9.25), (2.99, 5.80, 9.75), (3.30, 6.20, 10.25), (3.61, 6.60, 10.75), (3.93, 7.00, 11.25), (4.24, 7.40, 11.50), (4.56, 7.80, 11.75), (4.87, 8.20, 12.00), (5.19, 8.60, 12.25), (5.50, 9.00, 12.50), (5.81, 9.20, 12.50), (6.13, 9.40, 12.50), (6.44, 9.60, 12.50), (6.76, 9.80, 12.50), (7.07, 10.00, 12.50)}, while for *N*2 it is as follows: 

{(0.00, 0.00, 1.26), (0.00, 0.20, 1.76), (0.00, 0.40, 2.27), (0.00, 0.60, 2.77), (0.00, 0.80, 3.28), (0.00, 1.00, 3.78), (0.47, 1.80, 4.79), (0.93, 2.60, 5.80), (1.40, 3.40, 6.81), (1.87, 4.20, 7.82), (2.33, 5.00, 8.83), (2.64, 5.40, 9.33), (2.95, 5.80, 9.83), (3.27, 6.20, 10.34), (3.58, 6.60, 10.84), (3.89, 7.00, 11.35), (4.20, 7.40, 11.60), (4.51, 7.80, 11.85), (4.82, 8.20, 12.10), (5.13, 8.60, 12.36), (5.44, 9.00, 12.61), (5.76, 9.20, 12.61), (6.07, 9.40, 12.61), (6.38, 9.60, 12.61), (6.69, 9.80, 12.61), (7.00, 10.00, 12.61)}. 

In Step 7, the global fuzzy values of all packages which are between consecutive two limiting profiles are calculated. In the further part of this example, let us consider the package P=(10.5, 90, 1, A). In Table 8, one can find the fuzzy representations ${\tilde{p}}_{i}^{N1,\;}$, ${\tilde{p}}_{i}^{N2}$ of options from package P for both negotiators, *N*1, *N*2, obtained by using Formulas (16) and (17). 

Next, by using Formula (21) for fuzzy values from Table 7 and Table 8, we obtained the following fuzzy score of package P=(10.5, 90, 1, A):

$\tilde{F}\left(P^{N1}\right)=\left(2.54,\;3.79,\;5.83\right)$ for negotiator N1,

$\tilde{F}\left(P^{N2}\right)=\left(4.26,\;6.41,\;8.91\right)$ for negotiator N2.

In Step 8, each of the fuzzy representations of 5580 packages is compared with all 26 fuzzy limiting sub-profiles by using Formulas (18) and (19), for both negotiators. The values of $\mu_{P}$ for P=(10.5, 90, 1, A) are the following:

[1.00, 1.00, 1.00, 0.97, 0.90, 0.84, 0.72, 0.62, 0.53, 0.44, 0.36, 0.33, 0.29, 0.25, 0.22, 0.19, 0.15, 0.12, 0.09, 0.06, 0.03, 0.00, 0.00, 0.00, 0.00, 0.00] for recipient (*N*1), and [1.00, 1.00, 1.00, 1.00, 1.00, 1.00, 0.94, 0.85, 0.76, 0.68, 0.60, 0.57, 0.54, 0.50, 0.46, 0.43, 0.40, 0.37, 0.34, 0.32, 0.29, 0.27, 0.25, 0.23, 0.20, 0.18] for supplier (*N*2).

According to the classification described in Step 8, the values of $\mu_{P}$ allow the package P to be classified into the appropriate category. An exemplary package P was assigned by the recipient to cluster no. 9, and by the supplier to cluster no. 13.

**Figure 6:**
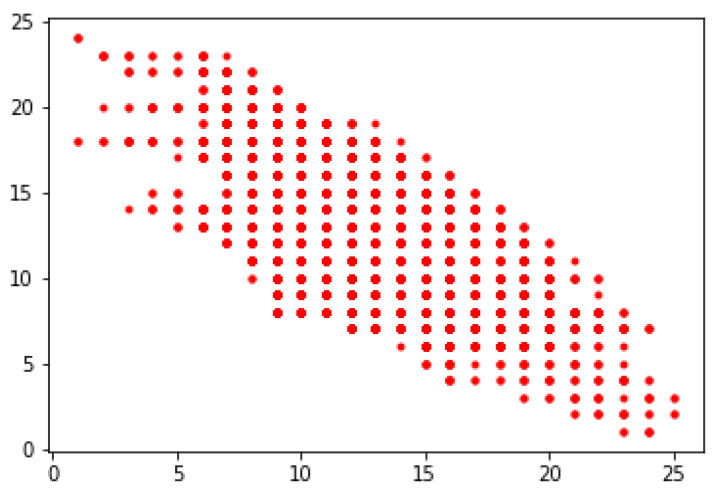
Negotiation space.

In Step 9, the obtained classifications for both parties are presented as the points of two-dimensional space (see Figure 6). The horizontal axis shows the cluster number assigned to the supplier, and the vertical axis shows the cluster number assigned to the recipient.

The first step in determining the best packages for both parties is to indicate the efficient frontier. Next, among the offers on this frontier, the best ones are those which are Pareto-efficient. The best packages for both *N*1 and *N*2 turn out to be the following ones presented in Table 9.

**Figure 7:**
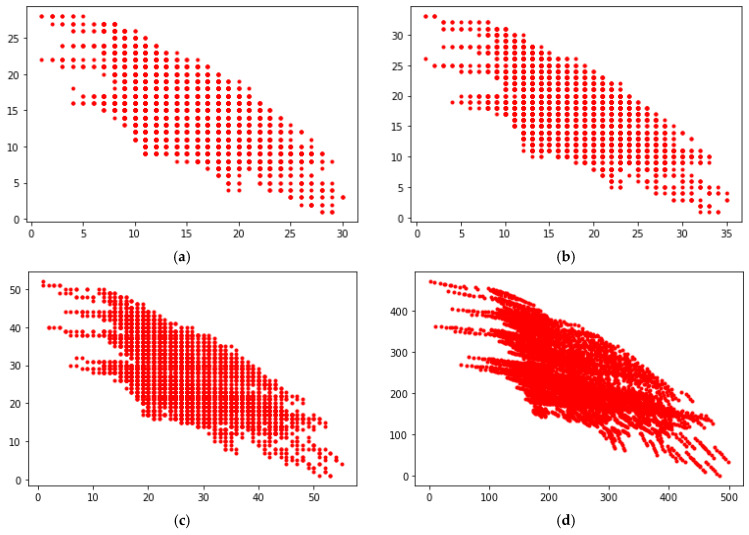
Negotiations space for various numbers of sub-profiles: (**a**) 31 sub-profiles; (**b**) 36 sub-profiles; (**c**) 56 sub-profiles; (**d**) 506 sub-profiles.

In the example shown, it is assumed arbitrarily that twenty-six limiting sub-profiles will be created in Step 6. It was also analyzed, as part of the model sensitivity analysis, whether a greater level of granularity of the division, i.e., the adoption of a greater number of limiting sub-profiles, will cause changes in the best packages for both *N*1 and *N*2. Figure 7 presents four cases in which the number of sub-categories was 5, 6, 10, and 100, respectively (in the above example, 4 sub-categories were used). This number of sub-categories generates 31, 36, 56, and 506 sub-profiles, respectively (see Figure 7).

In each of the analyzed cases, it turned out that, regardless of the increase in the number of limiting sub-profiles, exactly the same five packages turned out to be the best packages for both *N*1 and *N*2. Table 10 shows the results of the category determination for each of the five analyzed packages, assuming 31, 36, 56, and 506 sub-profiles, respectively.

When negotiators have the goal of choosing not a set but one absolutely best offer, then the number of fuzzy sub-profiles should be increased. In the analyzed empirical example, using ten sub-categories (56 sub-profiles) it was possible to choose the best package. Table 10 also presents the result of categorization of offers, assuming the presence of 506 sub-profiles. The differences between the individual packages became clearer. It was possible to develop a precise ranking of the packages. Apart from the second package, the fourth and the first package turned out to be the best.

## 7. Conclusions

The article presents a new fuzzy multi-criteria decision model for comprehensive negotiation support. The developed model can be a useful tool for the so-called third party in determining a set of effective and fair negotiation proposals. Thanks to the model, it is possible to take into account a wide variety of proposals while minimizing the information requirement. Moreover, the main advantage of the proposed model is that it can process linguistic information to minimize cognitive effort.

The main contribution of the authors relates primarily to three elements. Firstly, with the development of a new method of generating a set of negotiation issues on the basis of the sparse information obtained from negotiators expressed in fuzzy numbers. Secondly, with proposing a new scoring system for negotiation issues, and thirdly, with presenting a new method of generating reference set of limiting profiles. Our contribution is therefore both theoretical—in terms of developing a new model based on fuzzy numbers and practical—by developing a useful tool for managers to support the negotiation process. A limitation of the developed model is its bilateral nature. At this stage of the research, the authors did not assume the possibility of more than two parties to the negotiation. Another limitation of the model concerns the use of only triangular fuzzy numbers. Further research by the authors of the article will concern, on the one hand, the reduction of the current limitations of the model, i.e., the development of a new method by including the possibility of modeling the negotiation process in which more than two parties are involved. Moreover, it is assumed that fuzzy number concepts other than triangular ones can be used in the model. In addition, the authors plan to improve the determination of criteria weights in further studies. Currently, the model is based on simple linguistic scales. An interesting idea is to use other advanced weight determination methods such as Best Worst Method (BWM), Level Based Weight Assessment (LBWA), or Stepwise Weight Assessment Ratio Analysis (SWARA). Regarding the measurement scales used in the proposed model, it is important to investigate other types of measurement scales. This could be conducted using simulations. It would allow us to determine the relationship of the effect of a measurement scale on information granularity. It will also be a good idea in further research to compare the proposed model with methods based on rough theory, soft sets, or grey sets. Another important issue is that only a numerical example is shown in this paper. It is therefore advisable in the near future to study the use of the proposed method in the context of real negotiation problems. Real application of the model will allow us to validate its usefulness not only on the level of mathematical correctness but also in the context of business pragmatics. 

## Figures and Tables

**Table 1:** Linguistic terms for the weight ratings.

*L*	*LT*	*FTN*
1	Absolutely low important	(0.0, 0.1, 0.2)
2	Very low important	(0.1, 0.2, 0.3)
3	Low important	(0.2, 0.3, 0.4)
4	Medium low important	(0.3, 0.4, 0.5)
5	Medium important	(0.4, 0.5, 0.6)
6	Medium high important	(0.5, 0.6, 0.7)
7	Hight important	(0.6, 0.7, 0.8)
8	Very high important	(0.7, 0.8, 0.9)
9	Absolutely high important	(0.8, 0.9, 1.0)

**Table 2:** Linguistic terms for the option ratings.

*L*	*LT*	*FTN*
1	Very poor	(0, 0, 1)
2	Poor	(0, 1, 3)
3	Medium poor	(1, 3, 5)
4	Fair	(3, 5, 7)
5	Medium good	(5, 7, 9)
6	Good	(7, 9, 10)
7	Very good	(9, 10, 10)

**Table 3:** Negotiation template.

$\mathrm{Issues}\;\mathrm{to}\;\mathrm{Negotiate}\;(g_{i})$	$\mathrm{Options}\;(O_{i})$
Price (in US$) ($g_{1}$)	10; 10.5; 11; 11.5, …, 24.5; 25
Delivery time (in days) ($g_{2}$)	14; 21; 30; 45; 75; 90
$\mathrm{Payment}\;(\mathrm{in}\;\mathrm{days})\;(g_{3})$	1; 7; 14; 30; 45; 60
Returns conditions ($g_{4}$)	A; B; C; D; E ^1^

^1^ where: A—3% defects; no penalty, B—any defects; no penalty, C—7% defects; 4% penalty, D—5% defects; 2% penalty, E—5% defects; 4% penalty.

**Table 4:** Selected agreement packages.

$g_{i}\backslash P_{k}$	$P_{1}$	$P_{2}$	$P_{3}$	$P_{4}$	…	$P_{5577}$	$P_{5578}$	$P_{5579}$	$P_{5580}$
*g*_1_	10	10	10	10	…	25	25	25	25
*g*_2_	90	75	45	30	…	45	30	21	14
*g*_3_	1	1	1	1	…	60	60	60	60
*g*_4_	A	A	A	A	…	E	E	E	E

**Table 5:** Linguistic evaluation of the returns conditions’ options.

$g_{4}$	*N*1	*N*2
A	(0, 0, 1)	(8, 9, 10)
B	(0, 1, 3)	(5, 7, 9)
C	(5, 7, 9)	(3, 5, 7)
D	(3, 5, 7)	(0, 1, 3)
E	(8, 9, 10)	(0, 0, 1)

**Table 6:** The fuzzy weights for issues for *N*1 and *N*2.

$g_{i}$	$w_{i}^{N1}$	$w_{i}^{N2}$
$g_{1}$	(0.8, 0.9, 1.0)	(0.8, 0.9, 1.0)
$g_{2}$	(0.5, 0.6, 0.7)	(0.7, 0.8, 0.9)
$g_{3}$	(0.1, 0.2, 0.3)	(0.5, 0.6, 0.7)
$g_{4}$	(0.8, 0.9, 1.0)	(0.1, 0.2, 0.3)

**Table 7:** The fuzzy normalized weights for issues for *N*1 and *N*2.

$g_{i}$	${\tilde{w}}_{i}^{N1}$	${\tilde{w}}_{i}^{N2}$
$g_{1}$	(0.29, 0.35, 0.42)	(0.30, 0.36, 0.43)
$g_{2}$	(0.18, 0.23, 0.29)	(0.26, 0.32, 0.39)
$g_{3}$	(0.04, 0.08, 0.12)	(0.18, 0.24, 0.30)
$g_{4}$	(0.29, 0.35, 0.42)	(0.04, 0.08, 0.13)

**Table 8:** The fuzzy options’ scores of package.

$g_{i}$	${\tilde{p}}_{i}^{N1}$	${\tilde{p}}_{i}^{N2}$
$g_{1}$	(8.87, 9.94, 10.00)	(0.00, 0.25, 1.50)
$g_{2}$	(0.00, 0.00, 1.00)	(9.00, 10.00, 10.00)
$g_{3}$	(0.00, 0.00, 1.00)	(9.00, 10.00, 10.00)
$g_{4}$	(0.00, 1.00, 3.00)	(7.00, 9.00, 10.00)

**Table 9:** The best packages.

Package	$g_{1}\;$	$g_{2}\;$	$g_{3}\;$	$g_{4}\;$	Category
1	17.5	45	1	E	16
2	18	45	1	E	16
3	18.5	45	1	E	16
4	18.5	45	7	E	16
5	20	30	1	E	16

**Table 10:** The best packages for various numbers of sub-profiles (a–d).

Package	$g_{1}\;$	$g_{2}\;$	$g_{3}\;$	$g_{4}\;$	Category (a)	Category (b)	Category (c)	Category (d)
1	17.5	45	1	E	19	22	34	306
2	18	45	1	E	19	22	35	313
3	18.5	45	1	E	19	22	34	304
4	18.5	45	7	E	19	22	34	309
5	20	30	1	E	19	22	34	304

## Data Availability

The authors exclude this statement because the study did not report any data.
